# Spatiotemporal evolution characteristics and prediction analysis of urban air quality in China

**DOI:** 10.1038/s41598-023-36086-4

**Published:** 2023-06-01

**Authors:** Yuanfang Du, Shibing You, Weisheng Liu, Tsering-xiao Basang, Miao Zhang

**Affiliations:** 1 Mathematical Department, Tibet University, Lhasa, Tibet People’s Republic of China; 2 School of Economics and Management, Wuhan University, Wuhan, Hubei China; 3 School of Economics, Jiangxi University of Finance and Economics, Nanchang, Jiangxi People’s Republic of China

**Keywords:** Environmental sciences, Mathematics and computing

## Abstract

To describe the spatiotemporal variations characteristics and future trends of urban air quality in China, this study evaluates the spatiotemporal evolution features and linkages between the air quality index (AQI) and six primary pollution indicators, using air quality monitoring data from 2014 to 2022. Seasonal autoregressive integrated moving average (SARIMA) and random forest (RF) models are created to forecast air quality. (1) The study’s findings indicate that pollution levels and air quality index values in Chinese cities decline annually, following a “U”-shaped pattern with a monthly variation. The pollutant levels are high in winter and low in spring, and low in summer and rising in the fall (O_3_ shows the opposite). (2) The spatial distribution of air quality in Chinese cities is low in the southeast and high in the northwest, and low in the coastal areas and higher in the inland areas. The correlation coefficients between AQI and the pollutant concentrations are as follows: fine particulate matter (PM_2.5_), inhalable particulate matter (PM_10_), carbon monoxide (CO), nitrogen dioxide (NO_2_), sulfur dioxide (SO_2_), and ozone (O_3_) values are correlated at 0.89, 0.84, 0.54, 0.54, 0.32, and 0.056, respectively. (3) In terms of short-term AQI predictions, the RF model performs better than the SARIMA model. The long-term forecast indicates that the average AQI value in Chinese cities is expected to decrease by 0.32 points in 2032 compared to the 2022 level of 52.95. This study has some guiding significance for the analysis and prediction of urban air quality.

## Introduction

For more than 30 years, from the Reform and Opening-up to the first decade of the twenty-first century, China’s economy has continued to grow at a high rate. However, this has come at the cost of increasingly serious environmental problems. Air pollution is one of the most significant environmental problems in China. For example, in 2010, national sulfur dioxide emissions were 21.851 million tons, nitrogen oxide emissions were 18.254 million tons, soot emissions were 8.291 million tons, and industrial dust emissions were 4.487 million tons. In this context, the Ministry of Ecology and Environment of the People’s Republic of China issued the newly revised “Ambient Air Quality Standards” in 2012 and “Technical regulation for ambient air quality assessment (on trial)” in 2013. The main substances impacting air quality include both short-lived pollutants (such as PM_2.5_ and O_3_) and long-lived greenhouse gases (such as CO_2_ and CH_4_). Both are generated through energy consumption and should be treated in a coordinated way. In fact, since the initial emphasis on environmental protection in the Constitution of the People's Republic of China in 1978, China has implemented a series of measures to address air pollution and improve air pollution prevention and control policies. In 2013, the “Action Plan for Air Pollution Prevention and Control” (“Atmospheric Ten Articles”) was issued, setting governance targets and providing guidance. Regional cooperation mechanisms have also been strengthened to coordinate environmental governance. The government has successively issued policies such as the “Three-Year Action Plan for Winning the Battle for Blue Sky,” the “2019 National Air Pollution Prevention and Control Work Key Points,” the “Fourteenth Five-Year” National Cleaner Production Implementation Plan, and the 2035 vision target. At the 20th National Congress of the Communist Party of China (CPC), the concepts of harmonious coexistence between humans and nature, and green development were introduced to address the increasing public demand for environmental protection. These goals highlight the need to evaluate ambient air quality; study urban air quality changes and the spatial–temporal distribution characteristics of air pollutants; and predict future environmental air quality. The resulting insights can inform guidance for the public to take preventive measures to avoid air pollution, and can provide an important theoretical basis for relevant government departments to conduct prevention and control policies. These steps would help China actively respond to air pollution, rather than simply passively monitoring it.

The topic of monitoring, evaluating, and predicting ambient air quality conditions has been of great interest among scholars worldwide^1–3^. Air quality research in China mainly focuses in three areas. The first area of research includes air quality studies at different scales and in specific regions. For example, studies have analyzed the interannual variation characteristics of air quality in central and eastern China^4^, in typical northern cities^5^, and in typical towns in the north and south of the country^6,7^. Studies have also considered interannual variations in air quality^8^, have compared urban–rural air quality levels, and have analyzed air quality variations during significant festivals and events. The second area of research focuses on the factors influencing air quality. These factors are complex, and include pollutant factors^9,10^, Population density^11^, energy^12^, anthropogenic factors^13,14^, meteorological elements^15,16^, and socio-economic factors^17,18^. He et al. conducted a study using AQI, meteorological factors, and socio-economic data. That study found that climate conditions were the leading causes of air pollution in Hebei Province, while anthropogenic emissions were the primary factors contributing to severe air pollution in the same region^19^. The third area of research involves air quality prediction analysis, focusing on three main types of methods: latent forecasts^20^, numerical forecasts^21^, and statistical forecasts^22–24^. Statistical forecasting predicts future trends by analyzing statistical patterns of input–output information related to air pollution. This approach has gained the attention of many researchers because of its quick and simple features. Finally, the integrated algorithm Random Forest (RF) is a new machine learning paradigm, and has become popular because of its advantages of good robustness and high prediction accuracy.

The models and methods used in previous studies on the spatiotemporal evolution characteristics of urban air quality in China are relatively mature. However, few studies have analyzed and predicted air quality for multiple cities across China and for a longer observation periods. In addition, previous research focused primarily on predicting AQI values at specific historical moments but did not incorporate historical concentration values of the six major pollutants into their prediction analysis. To address this topic, this study analyzes the daily AQI and data on six major air pollutants from May 2014 to August 2022 for 388 major cities in 31 provinces in China. The study analyzes the characteristics of the spatial and temporal distribution of air quality in Chinese cities, the changing trends, and the correlation between the major pollutants with significant effects. Moreover, historical AQI values and concentrations of the six major air pollutants were used as independent variables to establish SARIMA and RF models and predict future development of urban air quality related indicators in China. The study results provide a scientific basis for relevant atmospheric environment monitoring and air pollution control departments and may help inform measures to improve future air quality.

## Materials and methods

### Data source and data pretreatment

The air quality data used in this study are from the China General Environmental Monitoring Station, a platform that publishes real-time national urban air quality data. A total of 1,050,590 daily air quality data points are used for this study’s analysis and modeling, representing data from May 13, 2014 through August 27, 2022, for 388 major cities in 31 provincial-level administrative regions in China (excluding Hong Kong, Macao, and Taiwan) in China. The available data include the AQI and concentrations of O_3_, PM_2.5_, PM_10_, SO_2_, NO_2_, and CO. The AQI is an essential comprehensive indicator reflecting the level of air quality of a city. It is calculated using the concentration of six principal pollutants and is correlated with the increasing severity of air pollution. In other words, larger AQI values indicate higher levels of air pollution, and smaller AQI values indicate lower air pollution levels. The AQI levels are divided into six grades, according to The Technical Provisions on Ambient Air Quality Index (for trial): excellent (0–50), good (51–100), mild pollution (101–150), medium pollution (151–200), heavy pollution (201–300), and serious pollution (301–500).

This study focuses on examining the spatiotemporal variation characteristics and trends of AQI using daily real-time and time-varying data. First, data are classified and summarized using the statistical analysis software PYTHON (Jupyter Notebook 6.3.0). The missing values are replaced using the average data of the corresponding cities.

### Research methods

#### Correlation analysis and descriptive statistical analysis

Correlation analysis is widely used to analyze air quality problems, and studies have shown that this approach can effectively identify the key factors influencing hazy weather and elevated PM_2.5_ concentrations. Therefore, this study uses correlation analysis to investigate the correlation between AQI and the six major pollutant concentration indicators, with the goal of exploring the causes for these correlations based on extensive studies. In addition, this study also provides a descriptive statistical analysis of the annual and seasonal variations of urban air quality in China and the provincial and municipal distribution characteristics. This provides a basis for subsequent predictions.

#### SARIMA model

The analysis of time series decomposition reveals that monthly data on air pollution-related indicators in major Chinese cities exhibit both long-term and seasonal fluctuations. Furthermore, the six pollutant concentration indicators are significantly correlated with the AQI values for significant cities in China. There may also be correlations among the six major pollutants. This indicates that there is multicollinearity among all factors. This does not satisfy the condition of mutual independence, making direct linear regression analysis inappropriate. To address this issue, this study applies time series and random forest regression models to analyze and predict AQI to address whether the condition of mutual independence is violated for the data set. First, the SARIMA model is established based on data characteristics of previous AQI data, with the goal of predicting AQI data in 2022.

The general form of the SARIMA model is $SARIMA(p,d,q)(P,D,Q)^{s}$, expressed as:

1 $$\Phi_{P} (L)A_{P} (L^{s} )(\Delta^{d} \Delta_{s}^{D} y_{t} ) = \Theta_{q} (L)B_{Q} (L^{s} )\mu_{t} ,$$

where $y_{t}$ is the time series; $\mu_{t}$ is a random term; $\Phi_{P} (L)$ denotes the autoregressive characteristic polynomial; *p* denotes the autoregressive maximum lag; $\Theta_{q} (L)$ denotes the moving average characteristic polynomial; and *q* denotes the moving average maximum lag. The term $A_{P} (L^{s} )$ is the seasonal autoregressive characteristic polynomial; s denotes the length of the seasonal period; *P* denotes the seasonal autoregressive maximum lag; $B_{Q} (L^{s} )$ denotes the seasonal moving average characteristic polynomial; *Q* denotes the moving average maximum lag; and *d* denotes the non-seasonal single integral order, which is the single integer difference. The term $\Delta_{s}^{D} y_{t}$ denotes the D times seasonal difference, and D denotes the order of the seasonal term, which represents the seasonal difference.

#### Random forest model

Past theoretical and empirical research has shown that AQI values in Chinese cities have clear spatial and temporal interactions. The magnitude of AQI values is influenced by the spatial interactions and by the cumulative effect of historical pollutant concentrations over time. This study establishes a random forest regression model to predict the AQI from a nonlinear perspective, combining different pollutant impact factors over time and using the six pollutant concentration indicators at historical moments as independent variables.

The random forest algorithm is a combinatorial model consisting of decision trees $h_{i} (x_{t} )$. The regression tree takes the mean value based on each terminal node as the overall prediction result. Thus, for the sample $x_{t} \in R^{j}$, j is the number of features and the random forest $\overline{h}(x_{t} )$ is the average of the predicted results of all subtrees $h_{i} (x_{t} )$, expressed as follows:

2 $$\overline{h}(x_{t} ) = \frac{1}{k}\sum\nolimits_{i = 1}^{k} {h_{i} (x_{t} )}$$

where k is the number of decision subtrees.

Before using the model for forecasting, we first evaluate the model's predictive performance. Model accuracy is generally determined using the mean absolute percentage error (MAPE), and root mean square error (RMSE), mean squared error (MSE), and mean absolute error (MAE). In addition, the goodness of fit (GOF) and explained variance score (EVS) are also commonly used to measure the strengths and weaknesses of forecasting methods. A combination of different parameters should be considered to measure the accuracy of the model’s prediction performance, to ensure an effective modelling outcome.

### Analysis of the results

Spatial and temporal evolutionary characteristics of urban air quality in China.

#### Annual analysis of air quality

The first step is to describe the overall distribution characteristics and trends of the daily average AQI values and the concentration values of the six major pollutants CO, NO_2_, O_3_, PM_10_, PM_2.5_, and SO_2_ for Chinese cities from 2014 to 2022. Table 1 and Online Resource 1 show the results of the time-series change analysis of these data. The charts visually indicate that the AQI, CO, NO_2_, O_3_, PM_10_, PM_2.5_, and SO_2_ data have relatively similar distribution characteristics, with significant fluctuations. Time trends significantly influence the series and indicate that the seasons influence the cyclical fluctuation trend. The AQI in 2021 decreased by 26.75% compared to 2015, while the reduction was 22.1% between 2016 and 2021; the specific figures are listed in Table 1. The other six primary pollution concentrations also decreased year by year.

**Table 1:** Overall national annual air quality from 2014 to 2022.

Years	AQI (N/A)	CO (mg m^−3^)	NO_2_ (μg m^−3^)	O_3_ (μg m^−3^)	PM_10_ (μg m^−3^)	PM_2.5_ (μg m^−3^)	SO_2_ (μg m^−3^)
2014	81.96	1.12	35.32	58.01	94.30	54.65	29.69
2015	77.20	1.08	30.08	56.25	87.64	50.37	25.70
2016	72.59	1.03	30.37	58.48	82.57	46.50	22.24
2017	70.82	0.96	31.19	63.42	80.09	44.08	18.32
2018	66.21	0.86	28.41	64.80	75.16	39.26	13.88
2019	62.24	0.79	27.55	62.73	67.40	37.18	11.35
2020	56.40	0.73	24.80	61.81	58.83	33.30	10.20
2021	56.55	0.68	23.95	61.60	62.94	31.68	9.42
2022	52.95	0.64	19.76	69.91	55.16	29.35	8.56

Table 2 shows the classified daily air quality by grade according to the year. The urban air quality in China hit an “Excellent” level at the following percentages of days in the sequential nine years from 2014 through 2022: 75.00%, 78.97%, 82.24%, 83.74%, 86.73%, 88.67%, 91.41%, 91.36%, and 92.42%, respectively. This indicates an increasing trend year-by-year. The percentages of days exhibiting heavy and serious pollution for the same nine sequential years are 2.46%, 2.97%, 2.48%, 2.21%, 2.02%, 1.601%, 1.115%, 1.37%, and 0.98%, respectively. This shows a decreasing trend year-by-year. In general, the air quality of most cities is rated Excellent, followed by Good, with only a certain proportion of days reporting light pollution. There are even fewer days classified as having moderate pollution or above. Although the proportion of days with air pollution in Chinese cities has been decreasing in recent years, the proportion is not small, and air pollution still should be actively managed and controlled.

**Table 2:** National overall air quality pollution levels from 2014 to 2022 (N = 1,050,590 data points).

Years	Cities*Days(n)	Air quality level					
		Excellent	Good	Mild pollution	Medium pollution	Heavy pollution	Serious pollution
2014	Days (42,317 = 189*224)	22,148	9588	7450	2088	884	159
	Percentage	0.52338	0.226576	0.17605	0.04934	0.02089	0.00376
2015	Days (131,874 = 364*362)	63,837	40,299	18,091	5726	3135	786
	Percentage	0.484076	0.305587	0.13718	0.0434	0.02377	0.00596
2016	Days (132,351 = 363*365)	62,707	46,136	15,290	4933	2576	709
	Percentage	0.47379	0.348588	0.1155	0.03727	0.01946	0.0054
2017	Days (132,802 = 365*364)	63,058	48,149	14,673	3992	2232	698
	Percentage	0.474827	0.36256	0.11049	0.03006	0.016807	0.0053
2018	Days (131,616 = 366*360)	60,283	53,863	11,491	3319	2,027	633
	Percentage	0.458	0.4092	0.08731	0.0252	0.015	0.0048
2019	Days (132,323 = 366*362)	60,621	56,704	9760	3120	1731	387
	Percentage	0.4581	0.4285	0.07376	0.0236	0.013	0.0029
2020	Days (132,918 = 366*363)	70,565	50,933	7705	2233	1186	296
	Percentage	0.5309	0.3832	0.0579	0.0168	0.009	0.002
2021	Days (134,341 = 371*363)	73,562	49,174	7716	2049	1136	704
	Percentage	0.5476	0.366	0.0574	0.015	0.0085	0.005
2022	Days (81,485 = 343*238)	48,663	26,647	4160	1214	562	239
	Percentage	0.5972	0.32702	0.05105	0.0149	0.00689	0.0029

**Figure 1:**
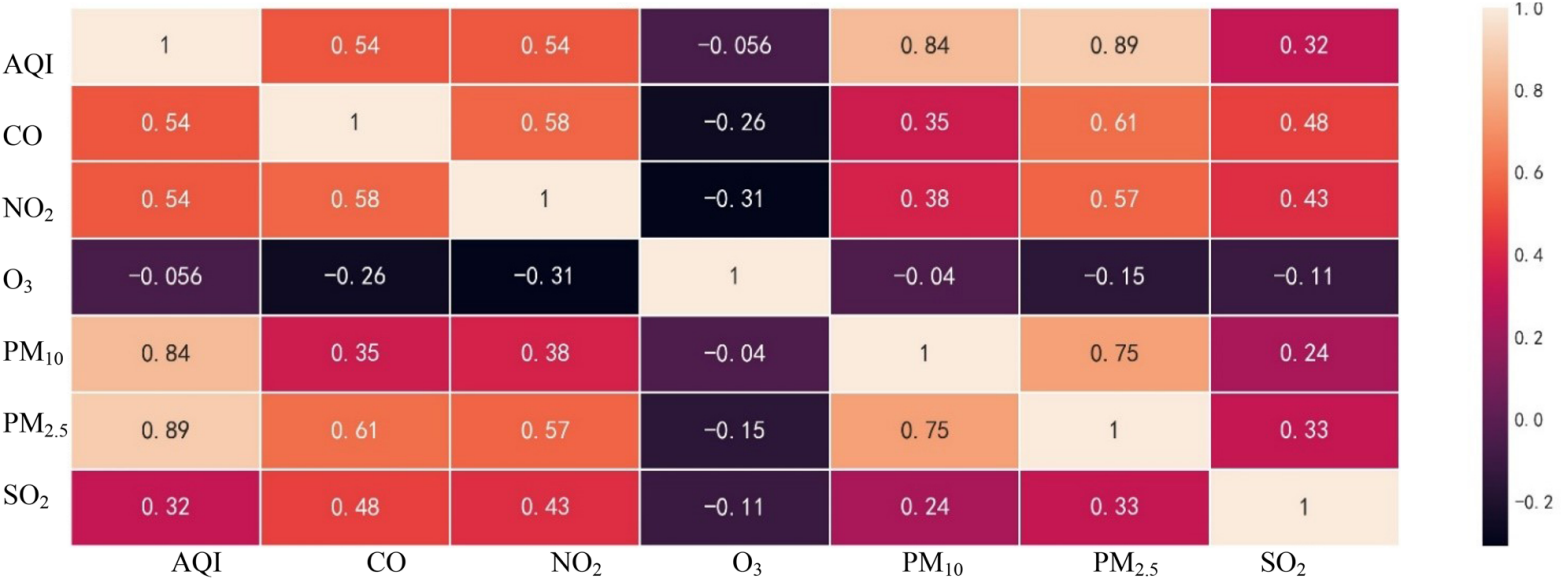
Heat map of AQI and six major pollutants in China.

The correlation coefficients between AQI and each of the following six pollutants, PM_2.5_, PM_10_, CO, NO_2_, SO_2_ and O_3_, are 0.89, 0.84, 0.54, 0.54, 0.32, and − 0.056, respectively (Fig. 1). The pollutant O_3_ is the only one with a negative correlation with AQI; all five other pollutants are positively correlated with AQI. Figure 1 shows that the increases in PM_10_ and PM_2.5_ concentrations are associated with the most significant increases in AQI. This may indicate that AQI is more sensitive to changes in particle concentration. Changes in ozone are mainly caused by solar radiation; as such, there is no strong correlation between changes in ozone concentration and changes in AQI. In addition, the correlation coefficients between the six pollutants, in particular between PM_2.5_ and PM_10_, PM_2.5_ and CO, and CO and NO_2_ concentrations, exceeded 0.58. Lang Lijun et al. also found that PM was strongly correlated with NO_2_, CO and O_3_-8h^25^. This indicates there is multicollinearity among all factors, highlighting the complexity of the correlation.

#### Seasonal analysis of air quality

In the comparative analysis, four seasons are divided according to the Gregorian calendar. As such, spring, summer, autumn, and winter are denoted as being March to May, June to August, September to November, and December to February, respectively. Table 3 shows the mean value of the AQI and concentrations of the six pollutants in the different seasons; the table indicates that the AQI and six pollutants in Chinese cities show significant seasonal variation. This result closely aligns with the findings of Ji Mengyi et al.^15^. In particular, the AQI in winter during the heating period is generally higher, with an average AQI of 86.64 (mild pollution). The overall AQI is lower in summer, with an average AQI of 47.62 (good). The results show that the air quality in Chinese cities is the worst in winter and the best in summer, due to seasonal variation in both natural and human activities. In winter, there is less dry precipitation, low temperature, stable air pressure, and temperature inversion. These conditions do not facilitate pollutant diffusion and dilution. As the heating season begins, pollutant emissions increase, exacerbating air pollution. In spring and autumn, the weather is mostly windy and sandy, affecting the ambient air quality. In summer, precipitation increases, humidity is high, and localized convection over the city is strong. This facilitates the deposition, dilution, and diffusion of pollutants, improving air quality.

**Table 3:** Data for air quality factors in different seasons.

Seasons	AQI (N/A)	CO (mg m^−3^)	NO_2_ (μg m^−3^)	O_3_ (μgm^−3^)	PM_10_ (μg m^−3^)	PM_2.5_ (μg m^−3^)	SO_2_ (μg m^−3^)
Autumn	61.89	0.86	30.23	54.28	69.20	37.68	15.49
Spring	67.96	0.79	26.67	72.64	81.54	38.67	14.54
Summer	47.62	0.70	19.77	75.04	48.83	25.23	11.07
Winter	86.64	1.14	35.02	43.21	93.94	60.18	22.88

Table 3 also shows that the PM_10_ and PM_2.5_ concentrations were highest in the winter season, and PM_2.5_, PM_10_, O_3_, and NO_2_ were highest in the spring season as the air quality indexes. O_3_ was highest in the summer season, likely because constant high temperatures and intense sunlight in summer tend to cause the photochemical reactions of nitrogen oxides and volatile organic compounds in vehicle exhaust and factory smoke emissions. This produces more ozone^26^. Heidarinejad et al. also reported that the highest number of unhealthy days associated with PM_2.5_ and PM_10_ pollutants occurs during the winter and spring seasons. However, their findings revealed that O_3_ levels are highest in winter, contradicting the conclusions drawn from our study^27^. Fang Lanlan et al. investigated the relationship between ozone (O_3_) concentration and the incidence of summer allergic skin diseases (ASD). Their study revealed a positive correlation between O_3_ concentration and hospitalization for ASD and chronic urticaria, providing indirect evidence of higher O_3_ concentrations during the summer compared to other seasons^28^.

**Figure 2:**
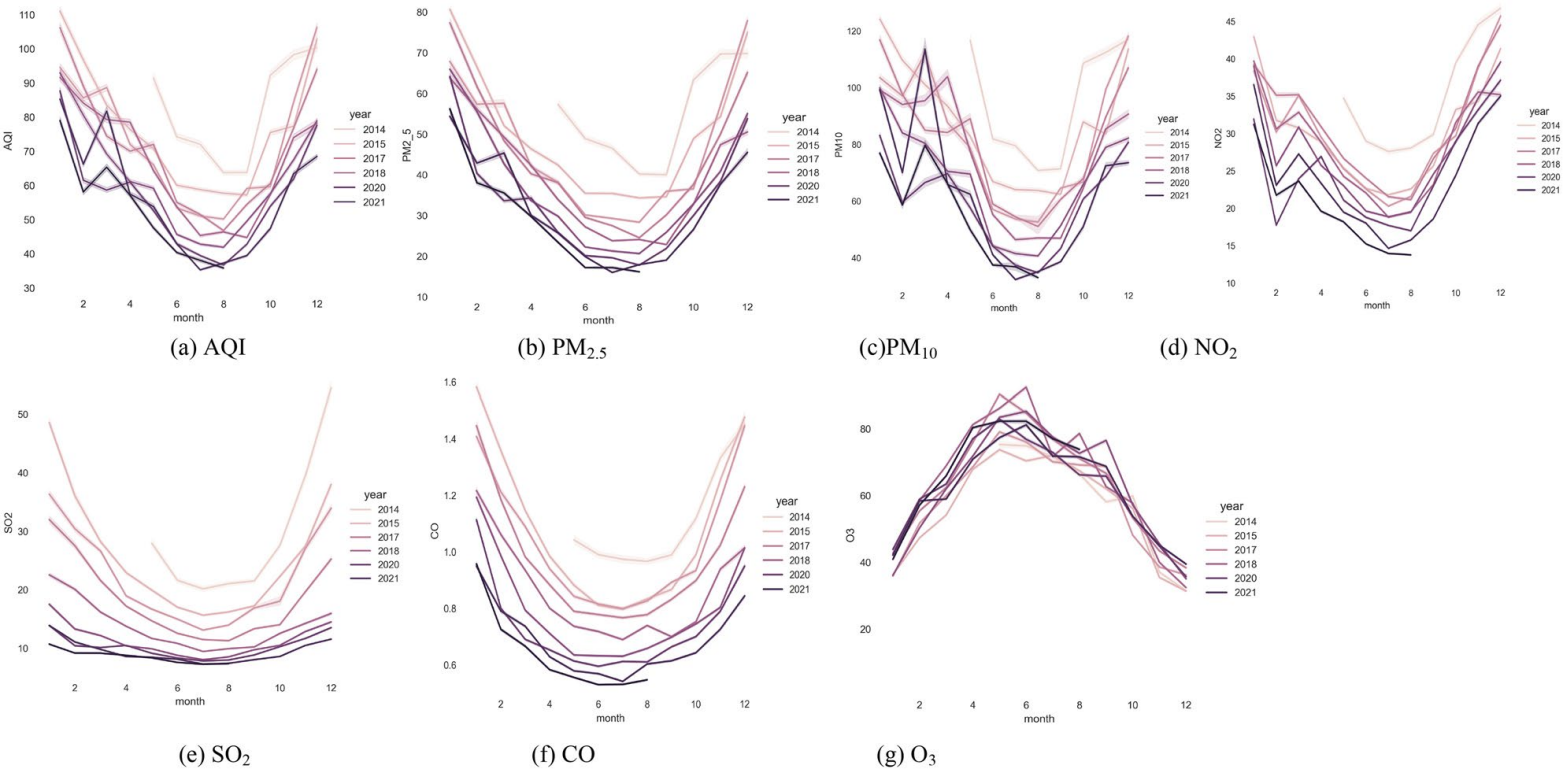
Monthly distribution characteristics of AQI value and concentration value of six pollutants.

Figure 2 shows the monthly data trend distribution of AQI values. The image visually shows that AQI is specifically related to the month, and there is a certain periodicity in the distribution of the monthly AQI. The monthly average AQI in 2014 is significantly higher than values in subsequent study years, especially in April, June, August, and November. The monthly average AQI values for 2019–2022 are significantly lower compared to 2014. Overall, the monthly average AQI value decreased continuously from March to July, reaching its lowest value from the end of July to the beginning of August. The value then gradually increased to the highest value in February of the following year. The AQI in Chinese cities shows a monthly “U”-shaped pattern of being high in winter, decreasing in spring, and then being low in summer, and rising in autumn. Among the six pollutants, five pollutants show a “U-shaped” distribution; only O_3_ has an “inverted U-shaped” distribution. This discovery provides valuable insights about the relationship between the air quality index and pollutants, which can inform the development of targeted air pollution control measures.

#### Provincial distribution of air quality

**Figure 3:**
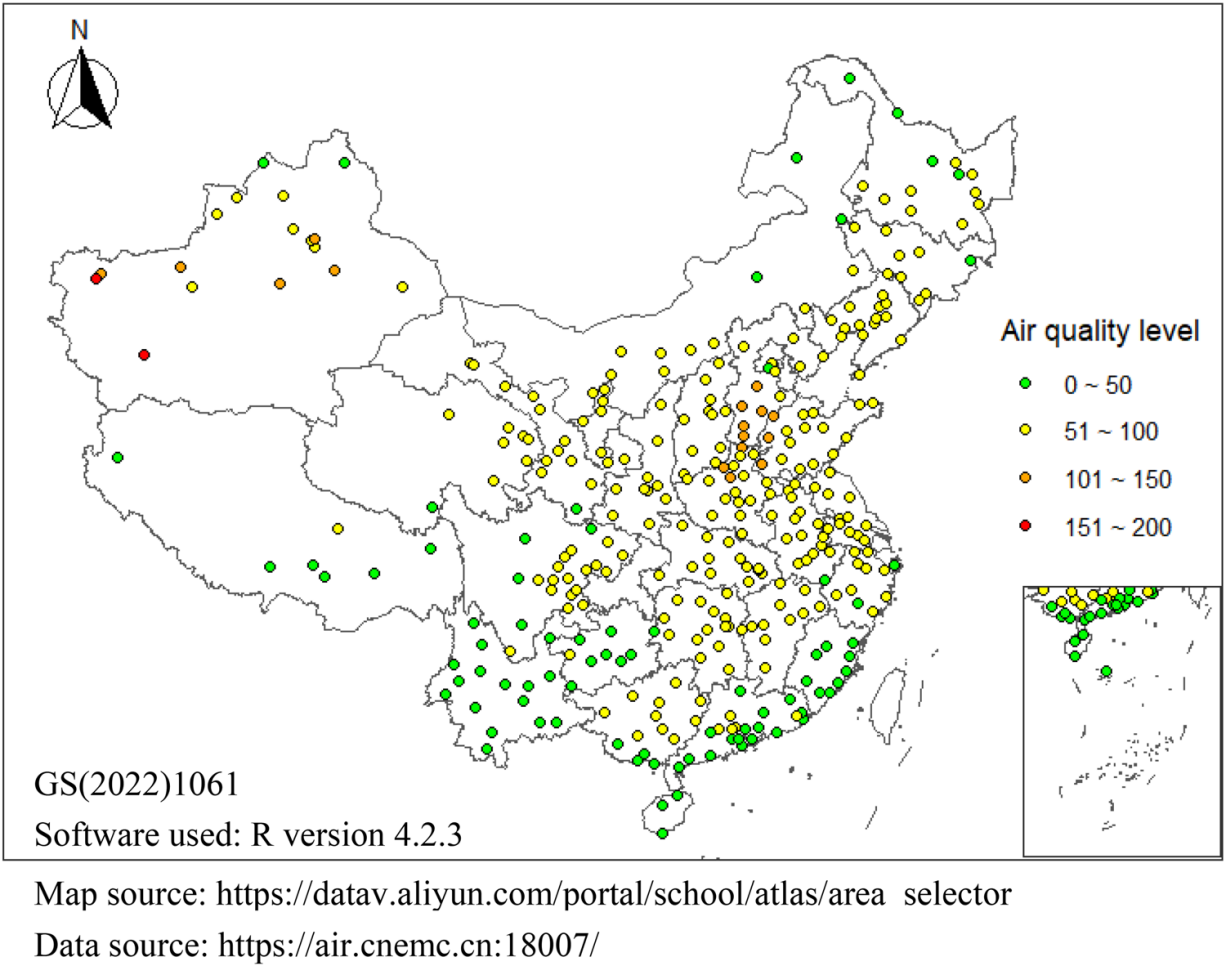
Spatial patterns of AQI in Chinese cities. *Note*: The map used in this study was generated based on the Alibaba Cloud Data Visualization platform, adhering to the GS (2022)1061 standard, with no modifications made to the base map boundaries. Data from Hong Kong, Macao, and Taiwan were not included.

Figure 3 shows the spatial distribution of AQI in Chinese cities from 2014 to 2022. The results indicate a significant lack of equilibrium with respect to the spatial distribution of urban air quality in China. The air quality is poorer in China’s central inland and northwestern regions, and is better in the southeastern coastal and highland areas. In general, the AQI of Chinese cities shows a spatial distribution pattern that is low in the southeast and high in the northwest, and low in the coast and high in the interior. These observations are largely consistent with the findings of Lin Xueqin and Wang Dai. (2016)^17^, as well as Wan Qing et al. (2022)^29^. This discovery holds significant reference value for gaining a comprehensive understanding of the regional disparities in urban air quality in China, and for conducting in-depth research into the root causes of air pollution. It also provides robust support for developing air pollution control strategies tailored to specific regions.

The AQI values of the 31 provinces are ranked and the ten provinces with the lowest AQI values are (ranked in order from lower to higher AQI values): Hainan, Xizang, Yunnan, Fujian, Guizhou, Guangdong, Heilongjiang, Guangxi, Qinghai, and Zhejiang. These ten provinces have satisfactory overall air quality and are free of air pollution. The ten provinces with the worst national air quality levels are (ranked in order from highest to lower AQI values): Henan, Xinjiang, Hebei, Tianjin, Shanxi, Beijing, Shandong, Shaanxi, Ningxia, and Hubei. The overall air quality of these 10 provinces is acceptable; however, some cities are more polluted than others, possibly impacting the health of susceptible people.

The primary pollutants in the ten provinces with the best air quality are PM_10_, PM_2.5_, and O_3_. The concentration levels of these three substances significantly influence the AQI values. This is particularly seen in the correlations between PM_10_ and PM_2.5_ and AQI, which exceed 0.94. Further, the correlation coefficient of the O_3_ concentration on AQI reaches 0.78. The correlation coefficient between PM_2.5_ and PM_10_ reaches 0.9; PM_10_ includes PM_2.5_, so an increase of PM_2.5_ also increases the PM_10_ concentration. The rise in PM_10_ cannot be smaller than the increase in PM_2.5_ concentration. As such, the correlation of 0.9 reflects reality. PM_10_ and PM_2.5_ are also the main pollutants in the ten provinces with the worst air quality.

#### Air quality municipal distribution

This study analyzes the air quality of 388 major cities in China based on the magnitude of AQI values. The ten cities with the best air quality are as follows (ranked in order of good to less good): Tibetan Autonomous Prefecture of Garzê, Linzhi, Danzhou, Sanya, Sansha, Tibetan Qiang Autonomous Prefecture of Ngawa, Yushu Tibetan Autonomous Prefecture, Qiannan Buyi and Miao Autonomous Prefecture, Altay Prefecture, and Diqing Tibetan Autonomous Prefecture. The ten cities with the worst air quality in the country are (ranked in order from poorest to better): Hotan Prefecture, Kashgar Prefecture, Aksu Prefecture, Kizilsu Kirghiz Autonomous Prefecture, Tulufan, Kuerle, Shijiazhuang, Anyang, Handan, and Xingtai.

**Figure 4:**
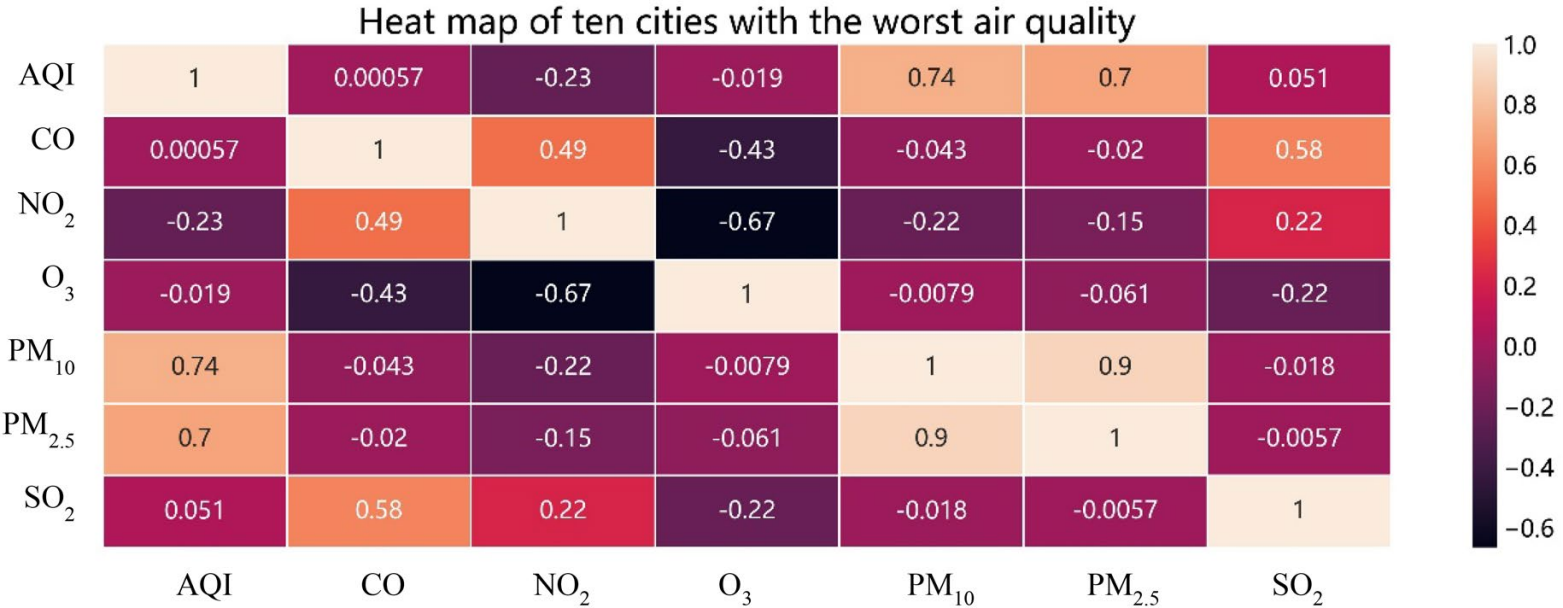
Heat map of the major pollutants in the ten cities with the worst air quality in China.

The main pollutants in the ten cities with the best air quality are PM_2.5_, PM_10_ and NO_2_; the correlation coefficients between these three pollutants and AQI are 0.76, 0.92, and 0.38, respectively. The correlation between PM_2.5_ and PM_10_ reaches 0.81; however, the other correlations among the six major pollutants are less than 0.37, and are not statistically significant. Figure 4 shows that CO, SO_2_, NO_2_ and O_3_ contribute little to the environmental air pollution of the ten most polluted cities. In contrast, PM_2.5_ and PM_10_ are the pollutant factors that most affect the environmental air quality of these cities. These pollutants are also closely correlated with urban air quality and provincial air quality. There is a strong positive correlation between PM_2.5_ and PM_10_, at 0.9, indicating that the increase of PM_2.5_ concentration accompanies the growth in PM_10_ levels.

### AQI prediction based on SARIMA model

#### Model parameter estimation

**Figure 5:**
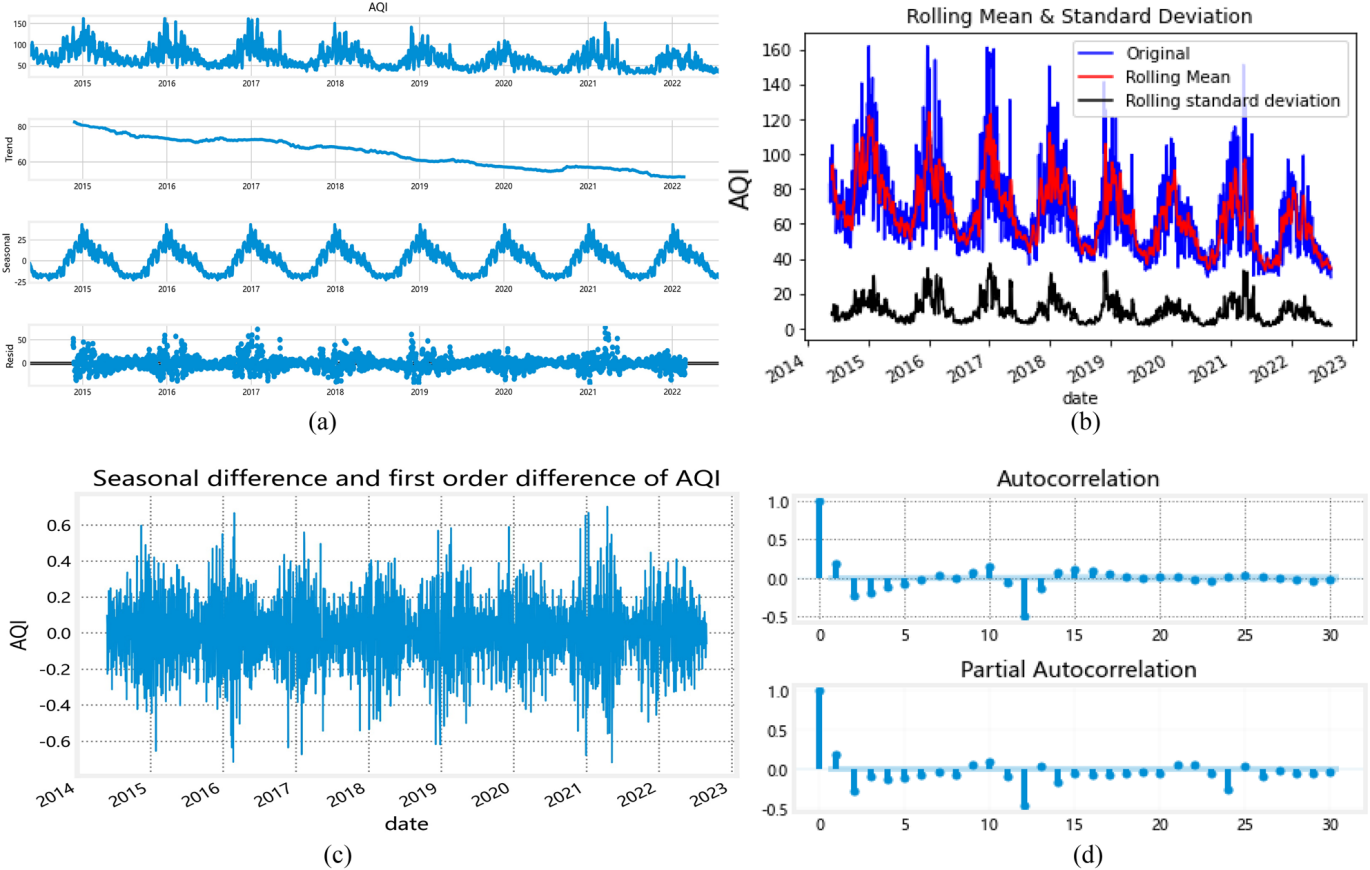
(**a**) AQI time-series diagram. (**b**) Moving average and weighted moving average of AQI. (**c**) Seasonal difference and first-order difference sequence diagrams of AQI. (d) ACF and PACF of mean monthly AQI after the seasonal difference and first-order difference.

First, we plot the AQI time series from May 2014 to August 2022 and decompose the time series directly into the trend and seasonal residuals to test for smoothness (Fig. 5). Figure 5 shows significant fluctuations in the AQI values for China from 2014 to 2022. The series appears to have a time-based trend, with a general decrease each year, and with significant seasonal characteristics. This indicates it is a non-stationary series. Therefore, this study generates a smooth non-white noise series by performing ordinary and seasonal difference operations on the original data (Fig. 5c,d). The smoothness is tested using the Augmented Dickey-Fuller test (ADF) method. The results are shown in Online Resource 2. The ADF statistical test results indicate that the hypothesized test values for the t-test to assess seasonal differencing and first-order differencing are less than the three critical values of 1%, 5%, and 10%.

For the modeling, this study uses a combination of Bayesian information criterion (BIC) and Akaike information criterion (AIC) statistics to determine the optimal order of the model. The BIC statistic is minimized by selecting different combinations of p and q parameters for repeated experiments and by combining the results generated by automatic screening using Python software. The model is determined to be $SARIMA(2,1,1)(0,1,1)^{12}$. The model parameters are provided in Online Resource 3.

#### Model fitting prediction

The SARIMA model equation is as follows:

3 $$(1 - 1.0029B + {0}{\text{.3404}}B^{2} )(1 - B)(1 - B^{12} )y_{t} = (1 - {0}{\text{.9196B}})(1 - 0.9909B^{12} )\mu_{t}$$

**Figure 6:**
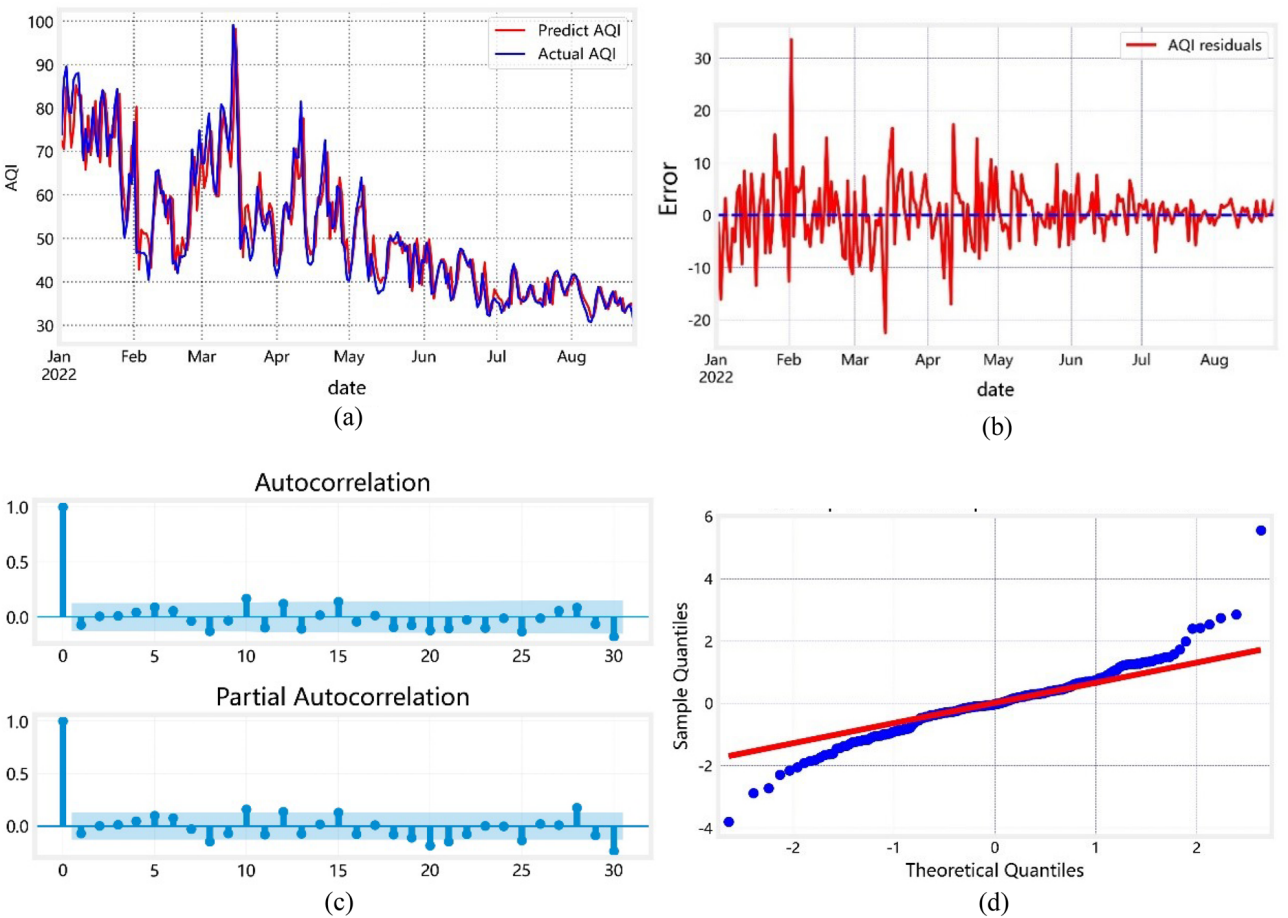
(**a**) The imitative effect of AQI simulated by $SARIMA(2,1,1)(0,1,1)^{12}$. (**b**) Residual diagram of SARIMA model. (**c**) Residual ACF and PACF after the seasonal difference and first-order difference. (**d**) Residual QQ Figure.

Figure 6 shows an overall good model fit, reflecting the trend of the monthly average AQI value for Chinese cities over a short time scale. The residual broken line diagram (Fig. 6b) indicates that the model is accurate, with some fluctuation in the residual difference between the predicted value and actual value. This trend is affected by the season. The deviation between the predicted and actual values may be due to inevitable errors in fitting the SARIMA model, based on the assumption there are no significant changes in other influencing factors. For example, the predicted value for February 2022 is slightly larger than the actual value, perhaps because the model does not consider the ban on fireworks during the traditional Chinese New Year.

A white noise test is performed on the residual series of the model to determine the model’s fitness. If the residual series falls within a white noise series, the model is considered to effectively explain the time series. Otherwise, the model needs to be further improved. The QQ chart in Fig. 6c shows that the residual series is normally distributed. The residuals pass the white noise test, indicating the extraction of useful information in the time series. The rest reflects random perturbation, which cannot be predicted and used. Therefore, the predicted values of the monthly AQI obtained from the model $SARIMA(2,1,1)(0,1,1)^{12}$ are closer to the actual situation, and the established model has an excellent fitting effect.

### Prediction of AQI values based on random forest model

#### Importance of random forests to assess pollution factors

The random forest algorithm is capable of predicting air quality from a non-linear approach, and can be used to both quantitatively and qualitatively analyze the specific relationships between the impact factors of pollutants and air quality and their degree of influence on AQI. To explore the importance of the six main pollutants, this study uses the constructed random forest model to select the important features of the pollutants affecting air quality.

This study uses the air quality grades from May 2014 to August 2022 as type variables. The AQI values and pollution factor data in the test set were entered into the trained RF prediction model to obtain the relative importance of each air pollutant concentration index. The relative importance when comparing concentrations of the six significant pollutants, PM_10_, PM_2.5_, CO, SO_2_, NO_2_, O_3_, and the AQI values are 39.69%, 32.28%, 13.04%, 8.80%, 5.37%, and 0.82%, respectively. The random forest model shows that PM_2.5_ and PM_10_ are the top two indicators that most significantly influence the AQI value. These are followed by CO, SO_2_, and NO_2_. These results are consistent with the results of the correlation coefficient analysis.

#### Forecast analysis of the random forest model

**Figure 7:**
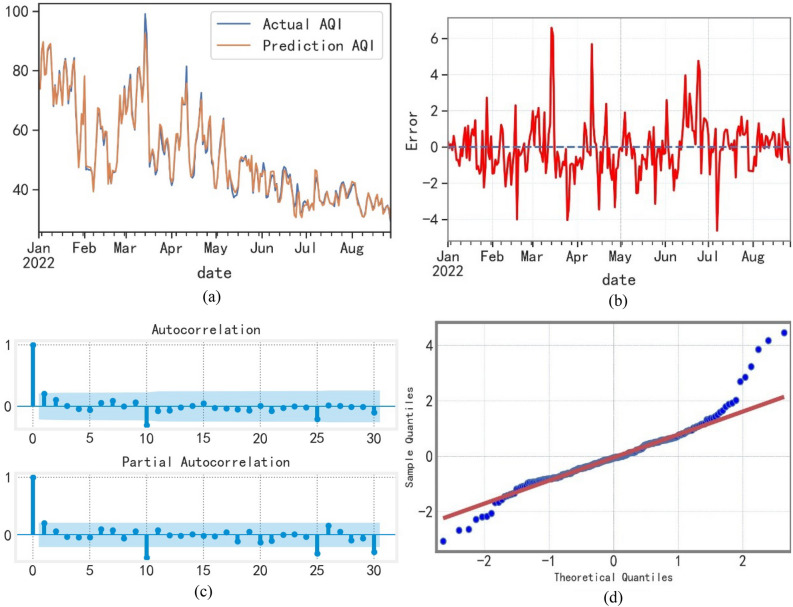
(**a**) Prediction curve of the Random forest regression model. (**b**) Prediction residuals diagram of the Random forest model. (**c**) Residual ACF and PACF after the seasonal difference and first-order difference. (**d**) Residual QQ Figure.

This study uses the average values of historical time-specific concentrations of six major pollutants (PM_2.5_, PM_10_, O_3_, NO_2_, CO, and SO_2_) from May 2014 to December 2021 as independent variables. The AQI values calculated from these pollution factors are used as dependent variables to construct a random forest model to predict AQI values for Chinese cities in 2022. Figure 7 shows the results.

Figure 7 shows that the predicted values are very close to the measured values, indicating a consistent trend and high prediction accuracy. However, certain factors (such as a sharp fall of temperature) cause a certain number of abnormal fluctuations in AQI. Because the random forest does not contain information about those factors, a certain amount of error is expected between the predicted value and the actual value.

A white noise test is performed on the residual sequence of the model to estimate the model’s suitability. The residual QQ shown in Fig. 7c indicates that the residual sequence passes the white noise test. The R^2^ of the random forest model is 97.61%; the MAE is 1.3841; the MAPE is 0.0228; and the EVS is 97.65%. This further indicates that the prediction accuracy is within a reasonable range and the model achieves a good fitting effect. In general, the variation trends with respect to the predicted and observed AQI values are highly consistent. This supports the conclusion that the regression model established using the RF algorithm performs well in predicting the AQI value.

#### Random forest prediction of future air quality

The empirical results show that the predicted values of both the SARIMA model and the RF model effectively match the trend associated with actual values, and achieve an effective benchmark for scale prediction. Model accuracy evaluation criteria were used to compare the fitting effect of the two models. The results are shown in Online Resource 4. The MAPE of the RF model and the SARIMA model are 0.0228 and 0.0951, respectively. The goodness of fit values are 0.976 and 0.662 for RF and SARIMA, respectively; and the RMSE values are 2.288 and 8.395 for RF and SARIMA, respectively. Based on these metrics, the RF model provides higher prediction accuracy, error rate, and reliability compared to the SARIMA model. This indicates that the random forest regression algorithm is effective in analyzing the effect size of each pollutant concentration on air quality, and in accurately predicting AQI index by pollutant concentration. The RF model’s validity and feasibility levels align with statistical laws and have practical significance.

Long-term scale forecasting helps analyze the air quality trends and patterns from a macroscopic perspective. Therefore, after verifying the feasibility and validity of the two models, this study applies the random forest model to develop long-term forecasts of the AQI and concentrations of the six study pollutants. The prediction results indicate that the average value of AQI in the next ten years is expected to be 51.09, with a minimum value of 29.48, and a maximum value of 137.84. This reflects a decrease compared to the average AQI of 64.99 from 2014 to 2022, and reflects a slight increase compared to the 2014–2022 minimum value of 29.21 and a slight decrease compared to the 2014–2022 maximum value of 161.88. Compared with 2020, the average AQI value for Chinese cities in 2032 is expected to decrease by 17.84. The mean concentrations of PM_2.5,_ PM_10,_ NO_2_, O_3_, SO_2_ and CO are expected to decrease by 17.08 μg m^−3^_,_ 56.57 μg m^−3^, 17.64 μg m^−3^, 47.04 μg m^−3^, 7.75 μg m^−3^, and 0.45 mg m^−3^, respectively. Of these, PM_10_, NO_2_ and ozone are expected to decrease most significantly. The forecast results indicate that the average air quality in Chinese cities is projected to further improve in the future. This is also consistent with the efforts of the government and people to improve air quality and control air pollution. The projections also indicate that the sharp decrease in pollutant concentrations, particularly with respect to aerosol particulate matter, may lead to a reduction in the cooling effect of particulate matter. This may hinder the expected mitigation of global warming. Therefore, it would be more appropriate to implement coordinated emission reduction measures that target both greenhouse gases and air pollutants, to achieve the goal of reducing global emissions.

### Ethics approval

This is an observational study.

### Ethical responsibilities

All authors have read, understood, and have complied as applicable with the statement on “Ethical responsibilities of Authors” as found in the Instructions for Authors and are aware that with minor exceptions, no changes can be made to authorship once the study is submitted.

## Conclusions and discussion

This research studies the temporal and spatial distribution characteristics of AQI and six major pollutants, using statistical analysis and correlation analysis methods, and time-based air quality monitoring data for 388 cities in 31 provinces of China from 2014 to 2022. The future air quality of Chinese cities is predicted using the SARIMA and random forest models. There were three key study findings:

1. There is a considerable downward trend in the AQI value and pollution concentration of Chinese cities overall across the study years. The AQI exhibits a “U”-shaped monthly trend that is high in winter and decreasing in spring, and low in summer and increasing in autumn. Summer generally has the best air quality and winter generally has the worst air quality (the pollutant O_3_ shows the opposite trend). Air quality in Chinese cities is spatially distributed as low in the southeast, high in the northwest, and low on the coast, and high in the interior.
2. Results indicate PM_2.5_ and PM_10_ are the principal pollutants in the provinces and cities in China with the worst air quality. Provincial and local authorities should pay close attention to SO_2_, CO, and NO_2_ emissions while concentrating on preventing and reducing PM_2.5_ and PM_10_ pollution emissions in the air. Pollution control practices should adhere to the principle of “prevention-oriented, combined with prevention and control” to promote the maintenance and continuous improvement of air quality. These pollutants are mainly caused by emissions from the burning of fossil fuels. As such, to mitigate and control air pollution, cities should adopt regional mitigation strategies to address air pollution in a coordinated manner. Actions taken by any single city to prevent and control air pollution are unlikely to be effective in a regional collection of heavily polluted cities. This highlights that air pollution management should not be restricted to a single city, and that a joint air pollution prevention and control approach is needed across administrative regions. Ultimately, an international system is needed to prevent and manage air pollution.
3. This study evaluates the importance of six significant pollutant variables on AQI using the random forest model. The results show that PM_10_ and PM_2.5_ remain the two pollutant indicators with the most critical influence on AQI. This is consistent with the results of the correlation analysis. Predicting the future AQI is a complex multivariate nonlinear problem, and both the SARIMA and RF models can predict AQI better than other models The prediction accuracy of the RF model is higher of the two, and the six pollutants' historical moment concentration variables may be more suitable than the AQI variables for air quality prediction with respect to the model training set. Experience has shown that environmental protection measures, such as road watering and a ban on lighting fireworks, have effectively controlled coarse particles and have successfully reduced particle concentrations, such as PM_10_ and PM_2.5_. It is also largely accepted that NO_2_, CO, and SO_2_ generally come from fuel ignition and engine vehicle fumes. In the future, the diminishing of these pollutant concentrations may mirror general commitment levels with respect to energy-saving and decreased emission approaches, such as the advancement of new energy vehicles in urban communities in the following 10 years.

This study’s statistical analysis and modeling methods have guiding significance for studies concerning air quality’s spatial and temporal evolution characteristics and future prediction. However, there remain many shortcomings and areas worth further research. When modeling the AQI influence factor analysis, this study did not consider the influence of meteorological elements, future economic development level, industrial structure, population change, and a series of policy interventions. Follow-up studier should consider the influence of more factors on air quality in China. In addition, applying a statistical-based approach is needed as an active research topic to establish the link between pollutant concentrations and AQI to predict air quality in future periods. Statistical methods are essentially based on historical data to make forecasts; as such, they have a significant advantage in multi-frequency short-term forecasting because the computational effort of statistical methods is several orders of magnitude smaller than required for numerical methods. However, the disadvantage of the statistical approach is that it requires a large amount of historical air quality data as the basis for model training to improve the prediction accuracy. With the advent of the Big Data era, traditional regression models are becoming obsolete, and machine learning—an interdisciplinary field of statistics and computer science is flourishing due to increased computing power. Studies such as Feng et al.’s work on using wavelet transform and artificial neural networks to predict PM_2.5_ highlight the potential of combining physical models and machine learning in air quality prediction^30^. The random forest algorithm is a prominent machine learning algorithm that is expected to evolve further and become a hot topic in big data processing algorithm optimization.

In closing, it is important to note that decreasing pollutant concentrations, especially the mass concentration of aerosol particles (PM), may reduce the cooling effect of the particulate matter. This may complicate the overall effort to mitigate global warming. Despite this, the temperature change caused by the sudden reduction in pollutant concentration is relatively small, and it is urgent to reduce greenhouse gases and air pollution around the world.

## Supplementary Information

Supplementary Information.

## Data Availability

The datasets analyzed for this study are located in the real-time national urban air quality release platform of the China General Environmental Monitoring Station. [https://air.cnemc.cn:18007/].
