# Supplementary material for: Spatiotemporal evolution characteristics and prediction analysis of urban air quality in China
Source: Sci Rep. 2023 Jun 1;13:8907. doi: 10.1038/s41598-023-36086-4 (PMC10235078; doi:10.1038/s41598-023-36086-4)
Supplement: Supplementary file 1 — Supplementary Information. [file 41598_2023_36086_MOESM1_ESM.docx]

Spatiotemporal evolution characteristics and prediction analysis of urban air quality in China

——Supplementary Material

Yuanfang Du*^1,2^, Shibing You^2^, Weisheng Liu^3^, BASANG Tsering-xiao*^1^ and Miao Zhang^2^

**^1^** Mathematical Department of Tibet University, Lhasa, Tibet, PR China. [ruyier521@whu.edu.cn (Y](mailto:ruyier521@whu.edu.cn%20(Y).D.); [basangtu@qq.com](mailto:basangtu@qq.com) (B.T.)

**^2^**School of Economics and Management, Wuhan University, Wuhan, Hubei, China. [00001839@whu.edu.cn](mailto:00001839@whu.edu.cn) (S.Y.); [zhangmiao123@whu.edu.cn](mailto:zhangmiao123@whu.edu.cn) (M.Z.)

**^3^**School of Economics, Jiangxi University of Finance and Economics, Nanchang, Jiangxi, PR China. [wilson.edu@gmail.com](mailto:wilson.edu@gmail.com)

^*^ Corresponding author. E-mail address: [ruyier521@whu.edu.cn](mailto:ruyier521@whu.edu.cn); [basangtu@qq.com](mailto:basangtu@qq.com)





***Online Resource 1.*** Time sequence diagram of AQI and six pollutants

***Online Resource 2.*** Augmented Dickey-Fuller test statistic.

|  | | | Test critical values | | |
| --- | --- | --- | --- | --- | --- |
| ADF | t-Statistic | p-value | 1% level | 5% level | 10% level |
| AQI | –2.61776 | 0.0894 | –3.4449 | –2.868 | –2.5702 |
| First order differential | –10.8446 | 1.58×10^–19^ | –3.4349 | –2.8636 | –2.5678 |
| Logarithmic AQI | –3.53195 | 0.0072 | –3.4346 | –2.8634 | –2.56778 |
| Seasonal difference | –8.83004 | 1.781×10^–14^ | –3.4347 | –2.8635 | –2.56779 |
| Seasonal 1st difference | –13.8 | 9.089×10^–26^ | –3.4347 | –2.8635 | –2.5678 |

***Online Resource 3.*** Parameters and test results of time series models

| **$SARIMA(p,d,q)(P,D,Q)^{s}$** | **$\varphi_{1}$** | **$\varphi_{2}$** | **$\theta_{1}$** | | **$\Phi_{1}$** | **$\Theta_{1}$** |
| --- | --- | --- | --- | --- | --- | --- |
| (2, 1, 1)(1, 1, 0)^12^ | 1.0463 | –0.3107 | ─── | | –1.0000 | –0.5088 |
| (2, 1, 1)(0, 1, 1) ^12^ | 1.0029 | –0.3404 | –0.9196 | | ─── | –0.9909 |
| **$SARIMA(p,d,q)(P,D,Q)^{s}$** | R^2^ | MAPE | RMSE | MAE | AIC | BIC |
| (2, 1, 1)(1, 1, 0) ^12^ | 0.5132 | 0.1993 | 18.35 | 15.0 | 10531.5 | 10557.89 |
| (2, 1, 1)(0, 1, 1) ^12^ | 0.4490 | 0.1794 | 14.92 | 12.44 | 10092.8 | 10119.13 |

***Online Resource 4.* The fitting effect of SARIMA model and Random forest model**

| Model evaluation | SARIMA model | Random forest model |
| --- | --- | --- |
| R^2^ | 0.6618 | 0.9761 |
| MSE | 70.4702 | 5.2328 |
| MAE | 6.3169 | 1.4757 |
| EVS | 0.5602 | 0.9748 |
| MAPE | 0.0951 | 0.0228 |
| RMSE | 8.3947 | 2.2875 |
